# Schmallenberg Virus in Domestic Cattle, Belgium, 2012

**DOI:** 10.3201/eid1809.120716

**Published:** 2012-09

**Authors:** Mutien-Marie Garigliany, Calixte Bayrou, Déborah Kleijnen, Dominique Cassart, Daniel Desmecht

**Affiliations:** University of Liège, Liège, Belgium

**Keywords:** Schmallenberg virus, bunyavirus, seroprevalence, cattle, Europe, Belgium, viruses

## Abstract

To determine prevalence of antibodies against Schmallenberg virus in adult cows and proportion of infection transmitted to fetuses, we tested serum samples from 519 cow/calf pairs in Belgium in spring 2012. Of cattle within 250 km of location where the virus emerged, ≈91% tested positive for IgG targeting nucleoprotein. Risk for fetal infection was ≈28%.

In the summer and fall of 2011, a nonspecific febrile syndrome, characterized by hyperthermia, drop in milk production, and watery diarrhea, was reported among adult dairy cows on farms in northwestern Europe (*1**,**2*). In addition, in November 2011, an enzootic outbreak emerged in several European countries; sequelae included abortion, stillbirth, and birth at term of lambs, kids, and calves with neurologic signs or malformations of the head, spine, or limbs (*3**,**4*). Both syndromes were associated with the presence in the blood (adult animals) or in the central nervous system (newborn animals) of the RNA of a new Shamonda-like orthobunyavirus, provisionally named Schmallenberg virus after the town in Germany where the first positive samples were identified (*3**,**4*). Because this new viral disease in cattle emerged recently, information on its epidemiology is limited. The objectives of this study were to determine the prevalence of antibodies against Schmallenberg virus in adult cows living within ≈250 km of the location where the virus emerged, 9 months after the emergence, and to determine the proportion of fetal transmission of the virus.

## The Study

**Figure 1:**
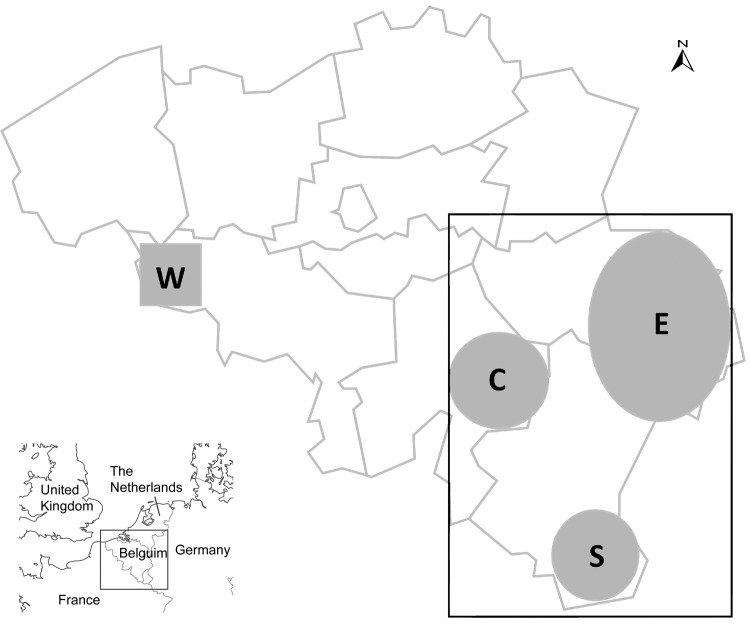
Location of 209 farms in Belgium from which 519 pairs of cow/calf serum samples were obtained from blood drawn by field veterinarians in southeastern (rectangle, 195 farms) or southwestern (square, 14 farms) areas in 2012. The area represented by the rectangle is centered on the village of Béthomont (50°08′N, 5°65′E) and measures ≈150 km from north to south and 100 km from east to west. Areas C (center), E (east), and S (south) refer to 3 distinct spatial clusters within the area represented by the rectangle. The area represented by the small shaded square (W) is centered on the village of Taintignies (50°54′N, 3°34′E); each side measures ≈20 km.

During February 13–April 22, 2012, serum samples were obtained at random from blood drawn by field veterinarians from 519 cow/calf pairs at 209 farms located in southeastern (195 farms; Figure 1, rectangle A) or southwestern (14 farms; Figure 1, square B) Belgium. Samples were obtained from 1–7 cow/calf pairs at each farm. None of the 519 calves exhibited neurologic signs of disease at birth through 10 months of age. Serum specimens were also obtained from a cohort of adult cattle in spring 2010 (n = 71) and the first quarter of 2011 (n = 40). We used the ID Screen Schmallenberg Virus Indirect ELISA kit (ID.vet Innovative Diagnostics, Montpellier, France) to determine if the serum samples contained IgG antibodies against the recombinant nucleoprotein of the emerging Schmallenberg virus. Results are expressed as percentages of the reference signal yielded by the positive control serum, with serologic status defined as negative (<60%), doubtful (>60% and <70%), or positive (>70%) by the manufacturer. Contingency tables were analyzed by using χ^2^ analysis to determine 1) if there was an association between sampling date and occurrence of seroconversion and 2) if there was an association between farm location and occurrence of seroconversion. Significance level was set at p<0.05.

**Figure 2:**
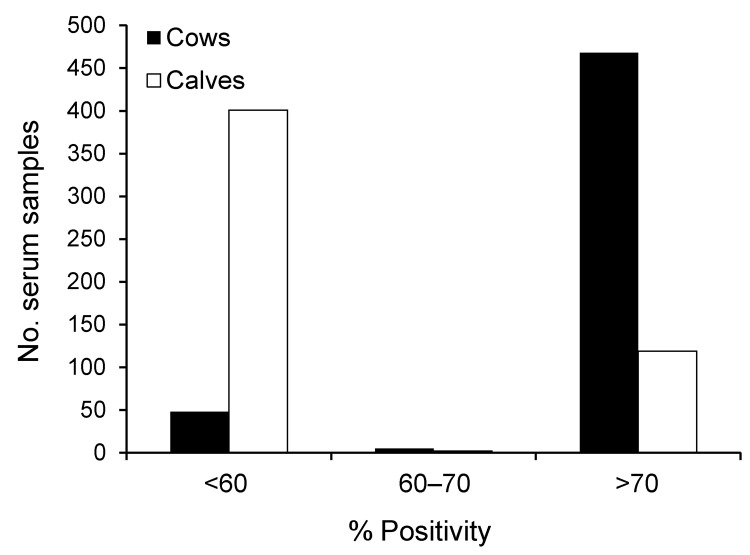
Frequency distribution of the results yielded by indirect ELISA for detecting IgG targeting recombinant nucleoprotein of the emerging Schmallenberg virus in serum samples from 519 cows, Belgium, 2012. Results are expressed as percentages of the reference signal yielded by the kit positive control serum, with serologic status defined as negative (<60%), doubtful (>60% and <70%), or positive (>70%) by the manufacturer.

All serum samples collected during spring 2010 and spring 2011 were negative for antibodies against Schmallenberg virus, which is consistent with the emergence of the new virus during the summer and fall of 2011 (*1*). Of the 209 farms sampled, only 13 were categorized as having seronegative cattle, each on the basis of the single paired sample that was available. These farms were not clustered by location. In each of the 196 remaining farms, >75% cows had seroconverted. Overall, apparent seroprevalence among adult cows was 90.8% (95%, CI 88.3–93.2, Figure 2). Association between farm location and seroconversion was not significant (p = 0.607), with results of 92.3%, 88.3%, 90.0%, and 92.0% in eastern, southern, western, and central areas, respectively (Figure 1). Acquired herd immunity against the new virus was thus quite high in the adult cattle population sampled, which suggests that this virus has spread quickly throughout the region since its emergence ≈250 km northeast of these areas in the late summer of 2011. Furthermore, a significant association between week of sampling and occurrence of seroconversion was found (p = 0.039), with a progressive increase of apparent seroprevalence: 87.8% (weeks 7–9, 95% CI 82.6–93.1), 90.4% (weeks 10–11, 95% CI 85.8–95.0), and 93.0% (weeks 12–16, 95% CI 89.6–96.4). This finding suggests that the virus was still circulating in the stables during the period examined. This results is not surprising because biting midges of the genus *Culicoides*, which are believed to transmit Schmallenberg virus (*5*), were recently shown to be able to complete their life cycle in animal enclosures (*6*).

Of the calves born to seropositive cows, 116 (24.6%) of 471 (95% CI 20.7–28.5; Figure 2) also tested positive, and no association was found between anti–Schmallenberg virus IgG in the newborn calves’ serum samples before they received colostrum and farm location (p = 0.639), with 23.8%, 23.0%, 31.6%, and 25.3% seroprevalence in eastern, southern, western, and central areas, respectively (Figure 1). Infection of pregnant cows by Schmallenberg virus thus often results in transmission to the fetus across the placenta, and this transmission does not lead automatically to abortion, stillbirth, or congenital deformities. Because none of the calves examined here showed the neurologic or musculoskeletal signs typically associated with unrestricted replication of Schmallenberg virus (*3**,**4*), maternal infection probably occurred after the fetuses became immunocompetent, that is, after the 150th day of gestation (*7*). Therefore, on the basis of the corresponding dates of artificial inseminations, we conclude that the 38 seropositive calves born during March 19–April 22, 2012, were infected after November 1, 2011.

Because the corresponding farms were not clustered spatially, we deduced that the new virus and its vectors actively circulated throughout southern Belgium in November 2011. Again, a significant association was detected between week of sampling and occurrence of seroconversion during gestation (p = 0.023), with a progressive decrease of apparent seroprevalence among calves born to seropositive cows: 28.4% (weeks 7–9, 95% CI 21.1–35.6), 21.0% (weeks 10–11, 95% CI 14.7–27.4), and 19.2% (weeks 12–16, 95% CI 13.9–24.4). Thus, the apparent rate of the virus crossing the placenta seems to decrease over time. Because the biology of the new virus would not have changed in a few weeks, this decreased rate of transmission could be attributed to the gradual increase in the relative proportion of cows that were infected before the development of the placenta enables placental transfer of the virus, i.e., before the 30th day of gestation (*7*). Therefore, to estimate the risk for fetal infection among immunologically naive cows, we focused on the group of seropositive cows whose gestation began long before the supposed emergence of the new virus. Of the 519 pregnancies, 148 resulted from artificial inseminations performed during May 2–22, 2011. The probability that the cows had been infected before the 30th day of gestation by a virus that emerged in late summer is close to zero. On the basis of data from this pertinent subset, we found that ≈28% of the calves born to cows primo-infected after the first placentome developed (which permitted the virus to cross the placenta) were indeed infected. This finding closely fits with the ≈30% reported for Akabane virus, a close phylogenetic relative of Schmallenberg virus (*7*).

In conclusion, this study confirms that the emerging virus was absent from the area examined in spring 2011 and provides evidence that 1 year later almost all adult cattle had seroconverted. Furthermore, the results suggest that the risk for infection of the fetus in an immunologically naive herd is ≈28% and that in utero infections can occur without sequelae visible at birth if the infection occurs when the fetus’s immune system is mature enough to control virus spread. In the case of Akabane virus, the cow’s natural immunity prevents subsequent infections of the fetus (*8*). It seems likely, therefore, that the Schmallenberg virus infection itself, and its resulting economic effects on farms in the regions concerned, might disappear in 2012.
